# Genotyped cluster investigations versus standard contact tracing: comparative impact on latent tuberculosis infection cascade of care in a low-incidence region

**DOI:** 10.1186/s12879-024-10358-4

**Published:** 2025-01-16

**Authors:** Michael Asare-Baah, Marie Nancy Séraphin, LaTweika A.T. Salmon-Trejo, Lori Johnston, Lina Dominique, David Ashkin, Krishna Vaddiparti, Awewura Kwara, Anthony T. Maurelli, Michael Lauzardo

**Affiliations:** 1 Department of Epidemiology, College of Public Health and Health Professions, College of Medicine, University of Florida, 2004 Mowry Road, PO Box 100231, Gainesville, FL 32610 USA; 2 Emerging Pathogens Institute, University of Florida, 2055 Mowry Road, PO Box 100009, Gainesville, FL 32610 USA; 3 Division of Infectious Diseases and Global Medicine, College of Medicine, University of Florida, 2055 Mowry Road, PO Box 103600, Gainesville, FL 32610 USA; 4 Institute of Public Health, Florida A & M University, Tallahassee, FL USA; 5 Florida Department of Health, Bureau of Tuberculosis Control, 4052 Bald Cypress Way, Bin A-20, Tallahassee, FL 32399 USA; 6 Department of Medicine, College of Medicine, University of Florida, Gainesville, FL USA; 7 Medical Service, North Florida South Georgia Veterans Health System, Gainesville, FL USA; 8 Department of Environmental and Global Health, University of Florida, Gainesville, FL 32610 USA

**Keywords:** Tuberculosis, Latent tuberculosis infection (LTBI), Cluster investigations, Contact investigations, LTBI Care Cascade

## Abstract

**Background:**

Cluster and contact investigations aim to identify and treat individuals with tuberculosis (TB) and latent TB infection (LTBI). Although genotyped cluster investigations may be superior to contact investigations in generating additional epidemiological links, this may not necessarily translate into reducing infections. Here, we investigated the impact of genotyped cluster investigations compared to standard contact investigations on the LTBI care cascade in a low incidence setting.

**Methods:**

A matched case-control study nested within a cohort of 6,921 TB cases from Florida (2009–2023) was conducted. Cases (*n* = 670) underwent genotyped cluster investigations, while controls (*n* = 670) received standard contact investigations and were matched 1:1 by age. The LTBI care cascade outcomes were compared using Pearson’s chi-square tests.

**Results:**

Of the 5,767 identified contacts, 3,230 (56.0%) were associated with the case group, while 2,537 (44.0%) were identified in the control group. A higher proportion of contacts were evaluated in the control group (85.5%) than in the case group (81.5%, *p* < 0.001). While the proportion of evaluated contacts diagnosed with LTBI did not significantly differ between the groups (case: 20.4%, control: 21.5%, *p* = 0.088), a higher percentage of LTBI-diagnosed contacts initiated TB preventive treatment (TPT) in the control group (95.9%) than the case group (92.9%, *p* = 0.029). TPT completion rates were similar, with 65.2% in the case group and 66.3% in the control group (*p* = 0.055). TB patients in the case group were more likely to be males, U.S.-born, Asians, residents of long-term care or correctional facilities, with past year histories of alcohol use, homelessness, and drug use.

**Conclusion:**

Despite the demographic and epidemiological differences between cases and controls, cluster investigations identified more contacts, with no significant difference in contacts diagnosed with LTBI, but were less effective than standard contact investigations in evaluating contacts, initiating LTBI treatment, and ensuring completion.

**Supplementary Information:**

The online version contains supplementary material available at 10.1186/s12879-024-10358-4.

## Introduction

Despite a reduction in tuberculosis (TB) incidence in Florida from 14.1 per 100,000 population in 1990 to 2.3 per 100,000 population in 2023, the goal of TB elimination (< 1 case per million population) remains distant [1]. Thus, efforts to interrupt transmission chains and prevent the reactivation of latent TB infections (LTBI) are critical [2].

In recent years, molecular genotyping of *Mycobacterium tuberculosis* (Mtb) has emerged as a valuable tool for TB control and surveillance, particularly in low-incidence and high-income settings [3, 4]. Conventional genotyping methods such as spoligotyping and mycobacterial interspersed repetitive unit-variable number tandem repeat (MIRU-VNTR) typing and, more recently, whole genome sequencing (WGS) have been instrumental in investigating TB outbreaks and transmission events [5–8]. Additionally, genotyping of Mtb is a valuable tool for program evaluation, as declining proportions of genotyped clusters over time provide a compelling metric for the effectiveness of TB control interventions [6, 7].

Utilizing Mtb genotyping in TB cluster investigations is a critical intervention that directly impacts the burden of the disease and the LTBI cascade of care. This approach helps to identify active cases for treatment and reduce the pool of LTBIs that can potentially result in active TB, especially among high-risk groups such as contacts of known cases [9–11]. In the US, cluster investigations may be initiated through multiple mechanisms. The CDC operates an outbreak detection algorithm that calculates the log-likelihood ratio (LLR) to identify higher-than-expected geospatial concentrations of a particular TB genotype within a specific jurisdiction. A higher LLR value signals a greater possibility of recent transmission, which can trigger a cluster alert [12, 13]. However, it is important to note that the LLR does not account for cases that are part of a cluster but have relocated to other areas of the state; these “missed contact” cases are still considered part of the cluster investigation. Alternatively, state and local TB programs may proactively create “Group Watch List” alerts for active surveillance when they observe a surge of a particular genotype in a specific location based on their surveillance reports [14]. These watch list clusters will generate alerts when a new case is added to the cluster. Both the CDC’s automated LLR-based alerts and the state-initiated watch list alerts are communicated to relevant health departments. Public health experts carefully review these reports to discuss clusters that may require further investigation or public health action [15].

Cluster and contact investigations represent two approaches to controlling TB transmission. Contact investigations focus on identifying and screening individuals who have been in close proximity to known TB cases, with the primary goal of detecting and treating active TB or LTBI among these contacts [16]. In contrast, cluster investigations employ Mtb genotyping to identify possible epidemiological links between cases with similar genotypes or single nucleotide polymorphisms (SNPs). This broader approach aims to uncover the transmission dynamics and potential outbreak sources beyond close contacts of known cases [17, 18]. While contact investigations are essential to promptly identify and manage individuals at elevated risk of infection from known TB cases, cluster investigations offer a more comprehensive understanding of transmission patterns within communities [19] by elucidating previously unrecognized transmission chains and interrupting ongoing transmission more effectively.

Cluster investigations have been shown to be more effective than conventional contact investigations alone in identifying and understanding TB transmission and uncovering epidemiological links that may be missed by contact investigations, which are largely limited to named contacts [20]. Despite this evidence, studies analyzing the LTBI care cascade in low-incidence countries, including the US, have revealed alarming gaps in implementing these investigations [9–11, 21]. These studies consistently demonstrated substantial attrition at each step of the cascade, from initial screening to treatment initiation and completion, highlighting missed opportunities to identify and treat at-risk contacts, ultimately hindering efforts to shrink the LTBI reservoir and prevent future TB cases.

Notwithstanding its utility over the past two decades, the epidemiological and population-level impact of cluster investigations as an intervention remains unknown. Here, we evaluated the effectiveness of genotyped cluster investigations in reducing the pool of LTBIs compared to standard contact investigations in a low incidence setting. We hypothesized that genotyped cluster investigations would significantly outperform standard contact investigations in identifying and treating individuals with LTBI.

## Methods

### Study design

**Fig. 1:**
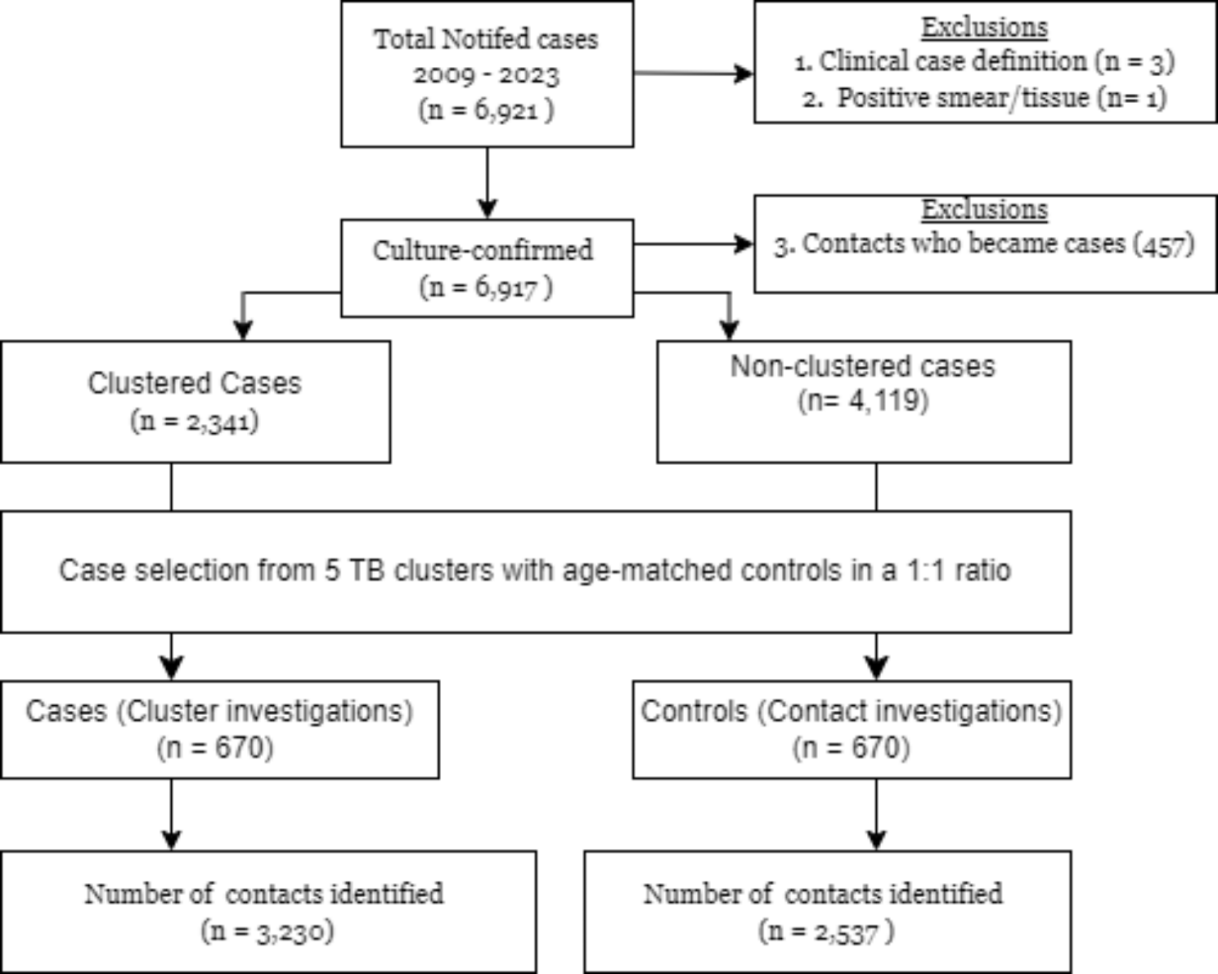
Study design and population

We conducted a matched case-control study nested in a TB cohort of 6,921 cases reported to the Florida TB program between 2009 and 2023 to assess the impact of TB cluster investigations on the LTBI cascade of care. To ensure accurate diagnosis, we only included culture-confirmed TB cases. We also excluded TB contacts who progressed to TB disease to avoid double counting these individuals as both cases and contacts (Fig. 1). TB cluster investigations were initiated when two or more cases met specific criteria: (1) shared identical or highly similar genotypes, and (2) demonstrated an established epidemiological link. Cases meeting these criteria were classified as clusters, while those with unique genotypes (singletons) underwent standard contact investigations following established protocols.

### Data sources

Three robust datasets linked to create a comprehensive picture of TB cases, and their contacts were used for this analysis. The primary data source was the Florida TB Registry, which contains detailed demographics, clinical and epidemiological information for all TB cases diagnosed in Florida, as captured in the Report of Verified Cases of TB (RVCT) [22]. This dataset was meticulously linked with genotyping information from the TB Genotyping Information Management System (TB GIMS), a secure web-based repository that manages genotyping data for all culture-confirmed TB cases in the US [23]. Finally, we linked both datasets with the contact/cluster investigation database, encompassing information on individuals evaluated as contacts for clustered and non-clustered cases. This additional layer captured crucial details regarding these contacts’ journeys through the LTBI care cascade, including screening, LTBI diagnosis, treatment initiation, and completion.

### Case definition

We defined our case group as 670 confirmed TB patients with established epidemiological linkage from five investigated TB clusters identified in Florida between 2009 and 2023. These clusters were identified using 24-locus MIRU-VNTR and confirmed by whole genome multilocus sequence typing (wgMLST). This purposive sampling represented the largest and most extensively investigated clusters, with complete data points on relevant variables.

### Selection of controls

We selected 670 patients as controls from a pool of non-clustered TB cases diagnosed during the same period as the case group that underwent standard contact investigations. Cases and controls were matched in a 1:1 ratio based on age to reduce selection bias and confounding and ensure comparability between the two groups, as different age groups have varying social behaviors or contact patterns associated with both the exposure (cluster investigation) and the outcome of interest (LTBI outcomes). This approach helped isolate the effect of cluster investigations on LTBI outcomes by reducing the impact of age-related differences between the groups.

### Exposure definitions

The exposure of interest was comprehensive cluster investigations, defined as in-depth investigations exceeding the scope of standard contact tracing protocols. This involved:

i. Enhanced data review: Thorough analysis of genotype and epidemiological data extracted from medical, laboratory, and other relevant sources to identify patterns and potential transmission pathways for all cluster cases.
ii. Expanded contact tracing: Interview patients and their contacts to map potential transmission chains and risk factors after initial standard contact investigations. This process goes beyond traditional close contacts to include leisure and social interactions, thus capturing a broader exposure network.

In contrast, the standard practice of contact investigation adhered to a concentric circle approach, relying primarily on the index case’s recall of close contacts (household members, friends, coworkers, classmates, and others) with limited medical record review.

### Outcome definitions

The following LTBI cascade outcomes were assessed.

- Number of contacts identified: This metric denotes the number of individuals potentially exposed to Mtb from a previously identified TB case. Verification was achieved by tallying the contacts. Each assigned a distinct identifier linked to a specific index case, as documented in the contact/cluster investigation register.
- Proportion of contacts evaluated: This metric shows the number of identified contacts who underwent screening for TB and LTBI. The evaluation was substantiated by records in the contact/cluster register that confirmed the screening process for each contact.
- Proportion of contacts diagnosed with LTBI: This indicates the number of contacts confirmed as having LTBI. LTBI diagnosis is derived from the contact/cluster investigation register, which consolidates diagnoses documented by healthcare providers. In cases where provider documentation is unavailable, an LTBI diagnosis is inferred for individuals who have commenced LTBI treatment.
- Proportion of contacts with LTBI initiated on therapy: This parameter quantifies the number of contacts who began tuberculosis preventive therapy (TPT). Initiation of therapy was confirmed through entries in the contact/cluster investigations register that recorded the commencement of treatment.
- Proportion of contacts with LTBI completing therapy: This metric represents the number of contacts who completed LTBI treatment. The register of contact/cluster investigations serves as the verifying source, with entries documenting treatment completion or otherwise for each contact.

### Variables of interest

To assess the differences between the two groups, we examined the influence of patient demographics: sex (male, female), birth origin (US-born, non-US-born), and race (White, Black, Asian, Other); clinical risk factors, such as patients’ HIV status, previous TB disease, any first-line anti-TB drug resistance, miliary TB, and cavitary TB; Epidemiological risk factors: past-year alcohol use, recreational drug use, homelessness, residence in long-term care, residence in correctional facilities, and occupation.

### Statistical analysis

A matching analysis was employed to create two groups with similar age distributions to address imbalances in the baseline characteristics. Participants were matched using the nearest neighbor technique with a caliper width of 0.2 in a 1:1 ratio. Matching was performed using the MatchIt package in R [24, 25].

Descriptive statistics, including proportions and standardized mean differences, were calculated to assess the distribution of the selected variables between cases and controls. Continuous variables were assessed using Student’s t-test, whereas categorical variables were examined using Pearson’s chi-square test.

The LTBI care cascade was constructed for the case and control groups by utilizing contact data for index cases that met the eligibility criteria and had completed contact/cluster investigations. Percentage frequencies and proportions were calculated to measure the cascade indicators at each step. Pearson’s chi-squared test was used to compare the two groups for each cascade indicator, with statistical significance defined as a *p*-value < 0.05. All analyses were performed using the R software version 4.3.0 (R Core Team, Vienna, Austria).

## Results

### Characteristics of study population

Our study population of 1,340 index TB cases revealed significant demographic and clinical characteristic differences between cases (patients offered cluster investigations; *N* = 670) and controls (patients offered contact investigations; *N* = 670). Cases had a higher proportion of males (68.1% vs. 59.0%, *p* = 0.001), were more likely to be born in the US (58.2% vs. 28.2%, *p* < 0.001), and had a higher percentage of Asians (24.7% vs. 17.2%, *p* < 0.001) than controls. Additionally, cases were more likely to reside in correctional facilities (5.4% vs. 1.3%, *p* < 0.001) and report higher rates of past-year alcohol use (21.0% vs. 8.7%, *p* < 0.001), homelessness (13.7% vs. 5.2%, *p* < 0.001), and recreational drug use (15.7% vs. 5.5%, *p* < 0.001) than controls. In contrast, the control group had a higher percentage of whites (43.2% vs. 33.2%, *p* < 0.001), Hispanics (27.3% vs. 10.7%, *P* < 0.001), and a higher prevalence of drug resistance (9.9% vs. 3.6%, *p* < 0.001). No significant differences were observed between the cases and controls regarding the site of disease, previous TB diagnosis, HIV status, cavitary TB, miliary TB, or occupation (Table 1).

**Table 1:** Demographic and clinical characteristics of the study population

Variables	Overall (*N* = 1340)	Cases (*N* = 670)	Controls (*N* = 670)	*P*-value
Sex = Male (%)	851 (63.5)	456 (68.1)	395 (59.0)	0.001
Race (%)				< 0.001
Asian	279 (20.9)	165 (24.7)	114 (17.2)	
Black	540 (40.5)	278 (41.6)	262 (39.5)	
White	509 (38.2)	222 (33.2)	287 (43.2)	
Other	5 (0.4)	4 (0.6)	1 (0.2)	
Hispanic = Yes (%)	255 (19.0)	72 (10.7)	183 (27.3)	< 0.001
Born In US = Yes (%)	579 (43.2)	390 (58.2)	189 (28.2)	< 0.001
Disease Site = Pulmonary (%)	1126 (84.0)	572 (85.4)	554 (82.7)	0.205
Previous TB Diagnosis = Yes (%)	42 (3.1)	24 (3.6)	18 (2.7)	0.433
Any Drug Resistance = Yes (%)	90 (6.7)	24 (3.6)	66 (9.9)	< 0.001
HIV positive (%)				0.667
Yes	177 (13.2)	85 (12.7)	92 (13.8)	
No	1049 (78.4)	532 (79.4)	517 (77.4)	
Unknown	112 (8.4)	53 (7.9)	59 (8.8)	
Cavitary TB (%)				0.283
Yes	526 (39.3)	260 (38.8)	266 (39.7)	
No	666 (49.7)	344 (51.3)	322 (48.1)	
Unknown	148 (11.0)	66 (9.9)	82 (12.2)	
Miliary TB (%)				0.502
Yes	52 (3.9)	23 (3.4)	(4.3)	
No	1121 (83.7)	568 (84.8)	553 (82.5)	
Unknown	167 (12.5)	79 (11.8)	88 (13.1)	
Resident Long-term Care Facility = Yes (%)	12 (0.9)	11 (1.6)	1 (0.1)	0.009
Resident Correctional Facility = Yes (%)	45 (3.4)	36 (5.4)	9 (1.3)	< 0.001
Past Year Alcohol Use (%)				< 0.001
Yes	199 (14.9)	141 (21.0)	58 (8.7)	
No	1136 (84.8)	527 (78.7)	609 (90.9)	
Unknown	5 (0.4)	2 (0.3)	3 (0.4)	
Past Year Homelessness (%)				< 0.001
Yes	127 (9.5)	92 (13.7)	35 (5.2)	
No	1189 (88.7)	564 (84.2)	625 (93.3)	
Unknown	24 (1.8)	14 (2.1)	10 (1.5)	
Past Year Recreational Drug Use (%)				< 0.001
Yes	142 (10.6)	105 (15.7)	37 (5.5)	
NO	1189 (88.7)	560 (83.6)	629 (93.9)	
Unknown	9 (0.7)	5 (0.7)	4 (0.6)	
Occupation (%)				0.351
Correctional Facility Employee	2 (0.1)	1 (0.1)	1 (0.1)	
Health Care Worker	28 (2.1)	14 (2.1)	14 (2.1)	
Migrant Seasonal Worker	5 (0.4)	3 (0.4)	2 (0.3)	
Not seeking Employment	20 (1.5)	14 (2.1)	6 (0.9)	
Other Occupation	312 (23.3)	159 (23.7)	153 (22.8)	
Retired	55 (4.1)	20 (3.0)	35 (5.2)	
Unemployed	753 (56.2)	379 (56.6)	374 (55.8)	
Unknown	165 (12.3)	80 (11.9)	85 (12.7)	

### Matching analysis

The baseline matching analysis revealed a precise balance between the case group and controls across all age categories, with a *p*-value of 1.000, indicating no significant difference in the age distribution of the two groups (Table 2).

**Table 2:** Baseline matching analysis of cases and controls

Baseline Parameter	Overall (*N* = 1340)	Cases (*N* = 670)	Controls (*N* = 670)	*P*-value
Age Categories (%)				1.000
0–15	4 (0.3)	2 (0.3)	2 (0.3)	
16–24	120 (9.0)	60 (9.0)	60 (9.0)	
25–44	376 (28.1)	188 (28.1)	188 (28.1)	
45–64	572 (42.7)	286 (42.7)	286 (42.7)	
65+	268 (20.0)	134 (20.0)	134 (20.0)	

### LTBI cascade analysis

**Fig. 2:**
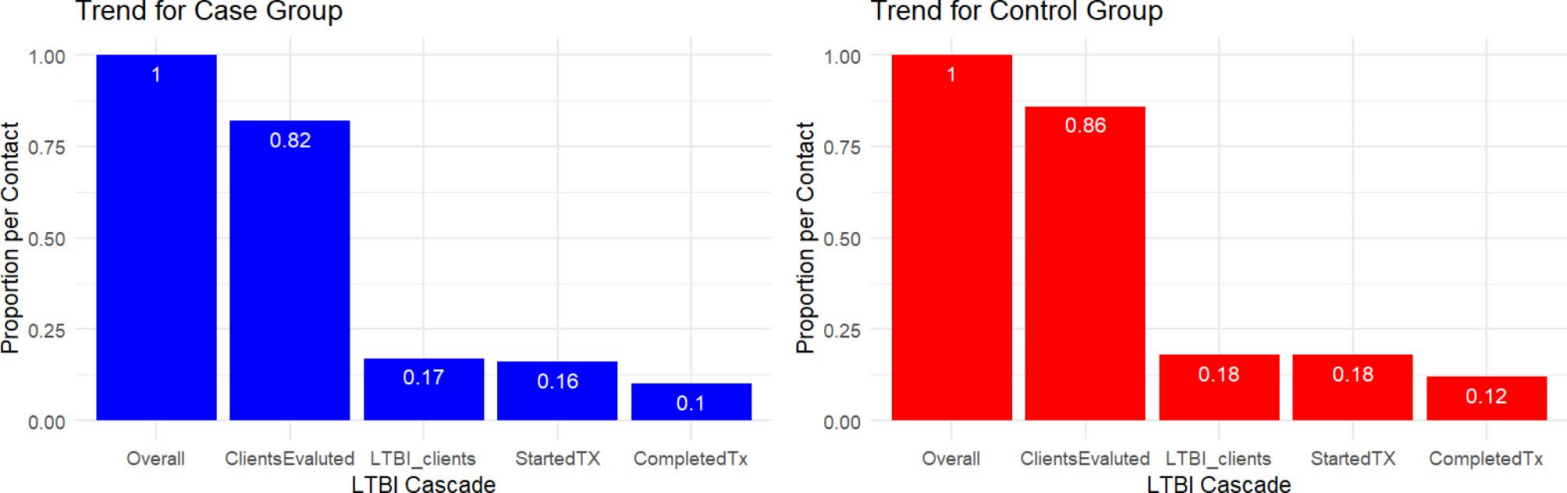
Comparison of cases and controls: proportion per contact

Among 1,340 TB cases in our study population, 866 were investigated, and 5,767 contacts were identified. Of these contacts, 4,800 (83.2%) were evaluated, with 73 (1.5%) diagnosed with active TB and 1,005 (20.9%) with latent TB infection (LTBI). Among LTBI-diagnosed contacts, 948 (94.3%) initiated TB preventive treatment (TPT), and 623 (65.7%) completed treatment (Table 3). Comparing cases who received cluster investigations with controls who were provided with standard contact investigations, a significantly higher proportion of contacts were evaluated in the control group (85.5%) than in the cases (81.5%) (*p* < 0.001). While no statistically significant difference was observed in the percentage of evaluated contacts diagnosed with LTBI between cases (20.4%) and controls (21.5%) (*p* = 0.088), a significantly higher proportion of LTBI-diagnosed patients in the control group (95.9%) initiated TPT than in cases (92.9%) (*p* = 0.029). TPT completion rates did not differ significantly between cases (65.2%) and controls (66.3%) (*p* = 0.055). The percentage of evaluated contacts who developed TB was slightly higher among cases (1.7%) than among controls (1.3%), but this difference was not statistically significant (*p* = 0.391) (Table 3; Fig. 2).

**Table 3:** LTBI cascade showing results of TB cluster/contact investigations

Outcome Indicators	Overall (*N* = 5767)	Cases (*N* = 3230)	Controls (*N* = 2537)	*P*-value
LTBI cascade				
Contacts evaluated (% contact)	4800 (83.2)	2632 (81.5)	2168 (85.5)	< 0.001
Diagnosed with LTBI (% evaluated)	1005 (20.9)	538 (20.4)	467 (21.5)	0.088
Initiated on TPT (% LTBI Diagnosed)	948 (94.3)	500 (92.9)	448 (95.9)	0.029
Completing TPT (% TPT Initiated)	623 (65.7)	326 (65.2)	297 (66.3)	0.055
TB Disease (% evaluated)	73 (1.5)	45 (1.7)	28 (1.3)	0.391

Notes: TPT (tuberculosis preventive treatment); LTBI (latent tuberculosis infection)

Each index TB case, on average, identified 6.7 contacts, with cluster investigations yielding slightly more (6.8) than contact investigations (6.4), and cluster investigations had a marginally higher evaluation rate 5.6 compared to contact investigations (5.5), with both groups having similar proportions of contacts diagnosed, initiated, and completing TPT (~ 1 per index case) (Supplementary Table 1).

## Discussion

This study aimed to investigate the effectiveness of genotyped cluster investigations compared with standard contact investigations in reducing the pool of LTBIs in a low incidence setting. Although genotyped cluster investigations yielded more identified contacts, surprisingly, the proportion of contacts evaluated for TB or LTBI and those initiating treatment for LTBI (TPT) was significantly lower than that of contact investigations. We observed similar proportions of contacts diagnosed with LTBI and those completing LTBI treatment for both interventions.

These results align with existing evidence in the literature, which generally suggests that genotyped cluster investigations are superior to standard contact investigations in identifying additional epidemiological links or “missed” contacts [17, 19, 26–28]. However, this may not translate to high proportions in subsequent steps of the LTBI care cascade for several reasons.

The timeliness of contact/cluster investigations is crucial for identifying and treating individuals with active TB and LTBI [29]. Standard contact investigations are typically initiated promptly after diagnosing an index TB case. However, the additional delays after the initial contact investigations process experienced in cluster investigations owing to the prerequisite procedure of culturing isolates, which may take 8–10 weeks, genotyping of the culture-confirmed isolates, and subsequent analysis to identify clustered cases could affect the increment in yield [27]. This delay can result in the loss of opportunities for public health action [17]. Delays in initiating cluster investigations could result in missing potentially infected contacts in a transmission chain through population migration before they are identified and evaluated.

Prioritization of contacts is a critical aspect of effective contact tracing for TB control [30, 31]. While both investigations examine close contacts, contact investigations prioritize evaluating individuals at the highest risk of infection and progression to active disease. This includes household members, coworkers, and individuals in common congregate settings who likely experienced prolonged exposure hours with the index case [31]. Such risk-based prioritization allows public health resources to focus on those contacts most likely to comply and adhere to LTBI screening and treatment initiation protocols. In contrast, cluster investigations cast a broader net to include casual contacts, as evidenced by the high number of contacts identified [31].

Additionally, genotyping data has inherent limitations, including not detecting all true transmission links, and may include additional cases with similar genotypes but not epidemiologically linked. They may also exclude clinically identified cases and cases that cannot produce sputum or have contaminated cultures, resulting in limited sampling [32–34]. This could diminish the incremental benefit of cluster investigations expected at subsequent steps of the LTBI cascade due to the potential inclusion of irrelevant contacts and exclusion of relevant ones.

The effectiveness of both investigations depends heavily on the overall public health infrastructure, resources, and implementation efficiency within a jurisdiction. Well-functioning TB control programs may achieve similar LTBI cascade outcomes regardless of investigation type. Overarching healthcare system factors like public health staffing, funding, and operational capacity potentially override the specific investigation strategy employed.

Moreover, social determinants impacting LTBI treatment initiation, adherence, and completion similarly affect clustered and non-clustered cases. Factors such as inadequate TB education, the stigma associated with the disease, lack of tact in conducting investigations by health staff, housing instability, substance use disorders, unemployment, limited social support, and the prolonged duration of preventive treatment regimens [35, 36] can undermine successful LTBI treatment in both clustered and non-clustered populations. Hence, in addition to cluster investigations, TB programs could address these fundamental social drivers, which are crucial for optimizing the population-level impact of TB control efforts, irrespective of the contact investigation approach.

The study acknowledges certain limitations, notably, the significant demographic and epidemiological differences between cases and controls. A substantial proportion of cluster investigation cases were male, U.S.-born, Asians, residents of long-term care or correctional facilities, with histories of alcohol use in the past year, homelessness, and drug use. While these factors may indicate a hard-to-reach population that could affect access to contacts, the effectiveness of contact investigations, and adherence to LTBI treatment protocols, they might also have resulted in more controlled oversight for tracking mobility and close contacts.

Individuals in institutional settings, such as long-term care and correctional facilities, have limited movement and well-defined networks of close contacts that are easier to identify. Additionally, those with substance use issues often receive care through integrated systems that can facilitate contact tracing.

The two groups exhibited comparability in terms of age, occupation, and disease characteristics, including pulmonary involvement, positive HIV status, miliary TB, and cavitary disease on chest imaging. These factors significantly influence TB transmission risk. While we report a low treatment initiation and completion rate among identified LTBI contacts for both investigation types, limited data on potential unmeasured patient, provider, or healthcare system-level barriers restricts our understanding of the underlying causes. Additionally, our findings are based on surveillance data from Florida, which may limit their generalizability to other settings.

In conclusion, while cluster investigations provide additional molecular epidemiologic insights, the core strategies of prompt evaluation, prioritization of high-risk contacts with LTBI for treatment, and ensuring treatment completion among those initiated on TPT, which drives the overall LTBI cascade effectiveness was lower compared to standard contact investigations.

Future research should focus on identifying factors that influence the effectiveness of the LTBI care cascade, such as healthcare system barriers, patient adherence, and socioeconomic determinants. Additionally, context-specific evaluations and tailored interventions may be necessary to maximize the impact of TB control strategies in different epidemiological settings.

## Electronic supplementary material

Below is the link to the electronic supplementary material.

Supplementary Material 1

## Data Availability

The dataset used in this analysis contains protected information from the national tuberculosis case report form and cannot be publicly shared to maintain patient confidentiality.
