# Supplementary material for: Genotyped cluster investigations versus standard contact tracing: comparative impact on latent tuberculosis infection cascade of care in a low-incidence region
Source: BMC Infect Dis. 2025 Jan 16;25:74. doi: 10.1186/s12879-024-10358-4 (PMC11740335; doi:10.1186/s12879-024-10358-4)
Supplement: Supplementary file 1 — Supplementary Material 1 [file 12879_2024_10358_MOESM1_ESM.docx]

**Supplementary Table 1: LTBI cascade showing yield per index case**

| **Outcome Indicators** | **Overall Index Case**  **(N= 866)** | **Cases**  **(N=472)** | **Controls**  **(N=394)** |
| --- | --- | --- | --- |
| LTBI cascade yield per index case |  |  |  |
| Contacts identified | 6.7 | 6.8 | 6.4 |
| Contacts evaluated | 5.5 | 5.6 | 5.5 |
| Diagnosed with LTBI | 1.2 | 1.1 | 1.2 |
| Initiated on TPT | 1.1 | 1.1 | 1.1 |
| Completing TPT | 0.7 | 0.7 | 0.8 |
